# Supplementary material for: Structural insights in cell-type specific evolution of intra-host diversity by SARS-CoV-2
Source: Nat Commun. 2022 Jan 11;13:222. doi: 10.1038/s41467-021-27881-6 (PMC8752678; doi:10.1038/s41467-021-27881-6)
Supplement: Supplementary file 1 — Supplementary Information [file 41467_2021_27881_MOESM1_ESM.pdf]

Supplementary Information

Structural insights in cell-type specific evolution of intra-host diversity by SARS-CoV-2

Kapil Gupta[1,2,†*], Christine Toelzer[1,2,†], Maia Kavanagh Williamson[3,†], Deborah K. Shoemark[1,2,†], A. Sofia F. Oliveira[1,4], David A. Matthews[3], Abdulaziz Almuqrin[3], Oskar Staufer[5,6,7,8], Sathish K.N. Yadav[1,2], Ufuk Borucu[1,2], Frederic Garzoni[9], Daniel Fitzgerald[10], Joachim Spatz[5,6,7,8], Adrian J. Mulholland[4], Andrew D. Davidson[3], Christiane Schaffitzel[1,2,10,*], Imre Berger[1,2,4,8,10,*]

[1] School of Biochemistry, University of Bristol, 1 Tankard's Close, Bristol BS8 1TD, UK

[2] Bristol Synthetic Biology Centre BrisSynBio, 24 Tyndall Ave, Bristol BS8 1TQ, UK.

[3] School of Cellular and Molecular Medicine, University of Bristol, University Walk, Bristol, BS8 1TD, UK.

[4] School of Chemistry, University of Bristol, Cantock's Close, Bristol BS8 1TS, UK.

[5] Department for Cellular Biophysics, Max Planck Institute for Medical Research, Jahnstraße 29, 69120 Heidelberg, Germany.

[6] Institute for Physical Chemistry, Department for Biophysical Chemistry, University of Heidelberg, Im Neuenheimer Feld 253, 69120 Heidelberg, Germany.

[7] Max Planck School Matter to Life, Jahnstraße 29, D-69120 Heidelberg, Germany.

[8] Max Planck Bristol Centre for Minimal Biology, Cantock's Close, Bristol BS8 1TS, UK.

[9] Imophoron Ltd, St. Philips Central, Albert Rd, St. Philips, Bristol, BS2 0XJ, UK.

[10] Halo Therapeutics Ltd, St. Philips Central, Albert Rd, St. Philips, Bristol, BS2 0XJ, UK.

† These authors contributed equally to this study.

* Corresponding authors: Kapil Gupta, 0044 (0)117 394 1251 kapil.gupta@bristol.ac.uk

Christiane Schaffitzel, 0044 (0)117 394 1869 cb14941@bristol.ac.uk

Imre Berger, 0044 (0)117 394 1857 imre.berger@bristol.ac.uk

Brief description of what this file includes:

Supplementary Fig. 1: Isolation of the wildtype and BriSΔ viruses.

Supplementary Fig. 2: SARS-CoV-2 WT and BriSΔ growth assays using MOIs determined on different cell types result in differences in infectivity.

Supplementary Fig. 3: Human convalescent serum efficiently neutralizes WT and BriSΔ viruses.

Supplementary Fig. 4: Purification and quality control of the BriSΔ glycoprotein.

Supplementary Fig. 5: Cryo-EM image processing workflow.

Supplementary Fig. 6: Cryo-EM structure validation.

Supplementary Fig. 7: BriSΔ RBD structural organization.

Supplementary Fig. 8: Masked 3D classification.

Supplementary Fig. 9: Spike proteins binding to ACE2 by Surface Plasmon Resonance (SPR).

Supplementary Fig. 10: Nonequilibrium simulations of wildtype S and BriSΔ proteins.

Supplementary Fig. 11: Average Cα-positional deviation in the five nanoseconds after removing LA from wildtype S protein.

Supplementary Fig. 12: Average Cα-positional deviation in the five nanoseconds after removing LA from BriSΔ protein.

Supplementary Fig. 13: Distributions of the distances between R408-V395, F377-Y369 and I434-L368 in WT.

Supplementary Fig. 14: Distributions of the distances between R408-V395, F377-Y369 and I434-L368 in BriSΔ.

Supplementary Fig. 15: Example of the FFA pocket in the beginning (t= 0 ns) and end (t=5 ns) of a nonequilibrium simulation of BriSΔ.

Supplementary Fig. 16: Distribution of the distance between R634 and Y837 in BriSΔ.

Supplementary Fig. 17: Number of hydrogen bonds between the R1039 and the protein in BriSΔ.

Supplementary Fig. 18: Distribution of the R1039-F1042 and R1039-E1031 distances in BriSΔ.

Supplementary Table 1: Cryo-EM data collection and refinement statistics.

Supplementary Table 1: Cryo-EM data collection and refinement statistics**.**

Supplementary Table 2: N-linked glycosylation sites in recombinant SARS-CoV-2 S proteins.

Supplementary Table 3: Alignment of SARS-CoV-2 S protein sequences used in this study

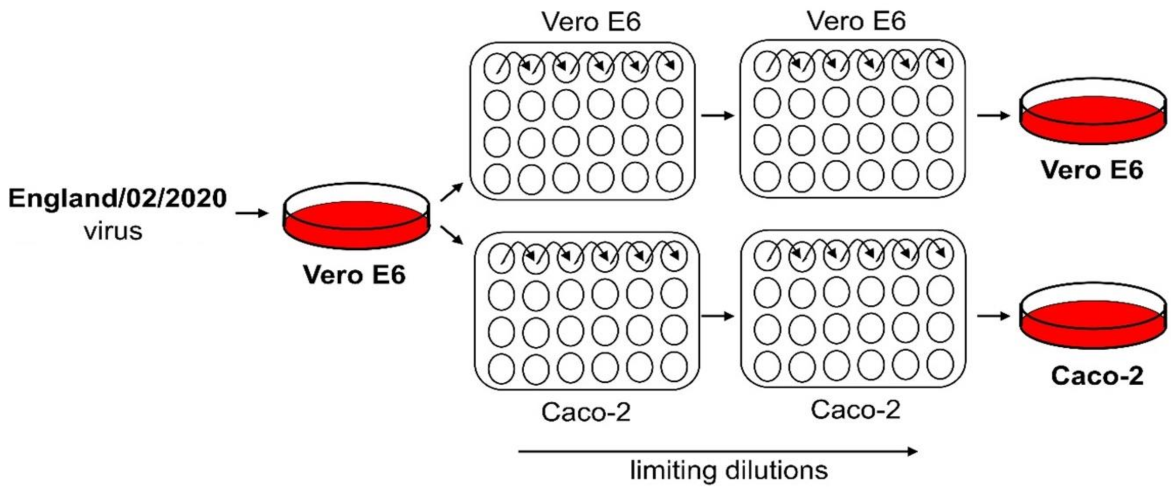

Supplementary Fig. 1: Isolation of the wildtype and BriSΔ viruses. Schematic illustrating how the mixed population of SARS-CoV-2 viruses with wildtype (WT) and the "Bristol" variant BriSΔ were separated from each other by two rounds of limiting dilution in different cell types. After initial growth of the mixed virus population in Vero E6 cells, the virus titer was determined by TCID50 assay and serially diluted in 96 well plates until statistically just one infectious virus particle was present in the wells with the highest dilution. In the wells with evidence of viral growth at the highest dilutions, the virus in the supernatant was assayed by site-specific PCR to determine if the virus was WT or the BriSΔ variant. This process was repeated to isolate a pure preparation of either virus which were then grown up to provide sufficient virus to sequence by dRNA-seq.

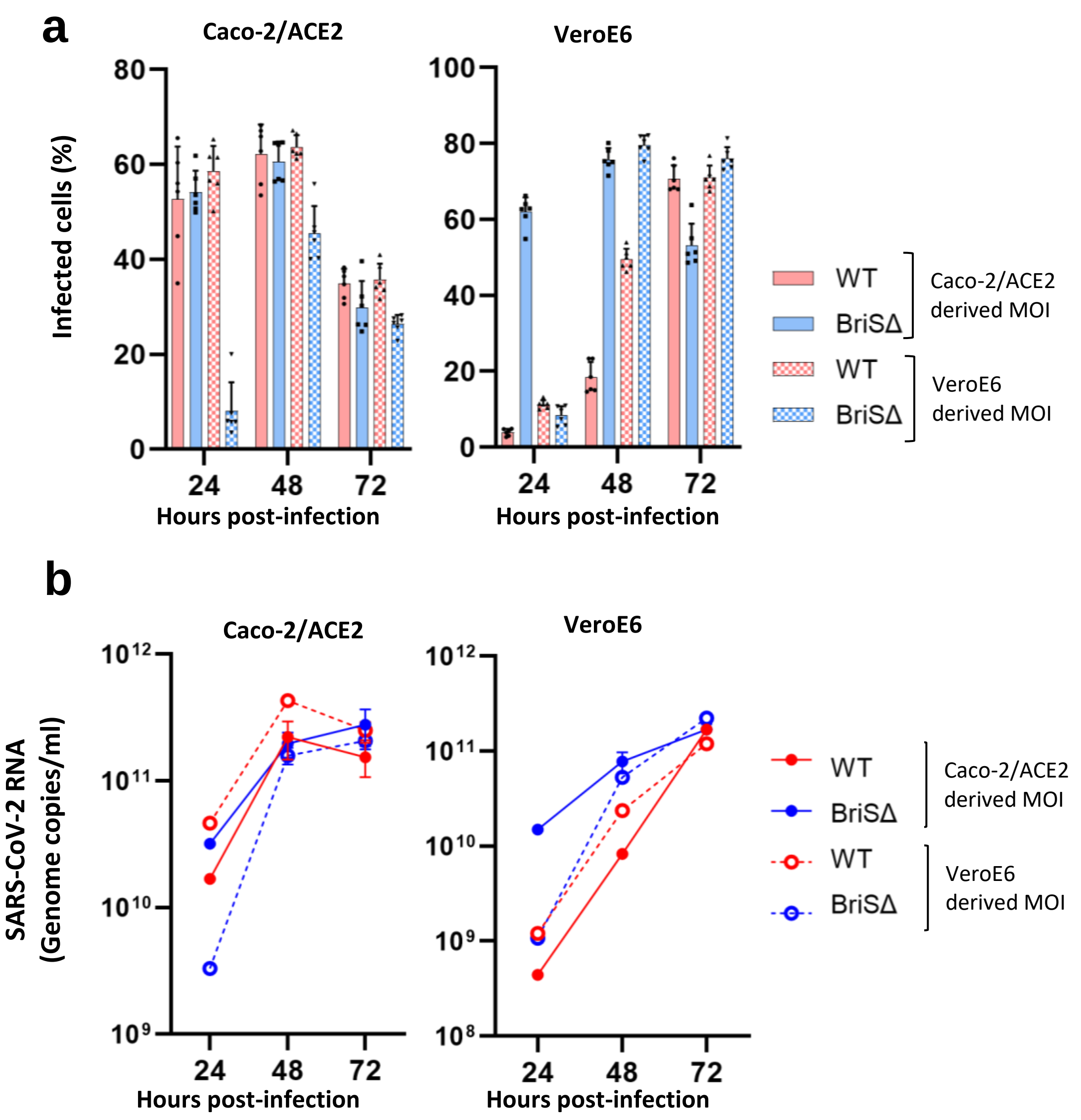

Supplementary Fig. 2: SARS-CoV-2 WT and BriSΔ growth assays using MOIs determined on different cell types result in differences in infectivity. Caco-2/ACE2 or Vero E6 cells were seeded in 96 well plates and infected with two different volumes of the WT and the BriSΔ virus stocks, each representing a MOI of 0.5 calculated from infectious titres previously determined on either Caco-2/ACE2 (red) or Vero E6 (blue) cells. At the indicated time points post-infection, supernatants were collected from the infected cells, which were then fixed in 4% PFA. **a.** The % of virus infected cells was determined by staining the fixed cells using an antibody against the SARS-CoV-2 N protein and DAPI to detect nuclear DNA, before analysis using the ImageXpress Pico automated imaging system. The data points for n=6 biological

replicates are overlaid on box-plots with the top of the box plot showing the mean values and error bars shown as + SD. **b.** Viral RNA was extracted from pooled culture supernatants from each replicate time point and the amount of viral RNA quantitated by qRT-PCR and expressed as genome copies/ml using a calibrated reference standard. qRT-PCR assays were done in technical triplicate. Data are presented as mean values with error bars showing +/- SD.

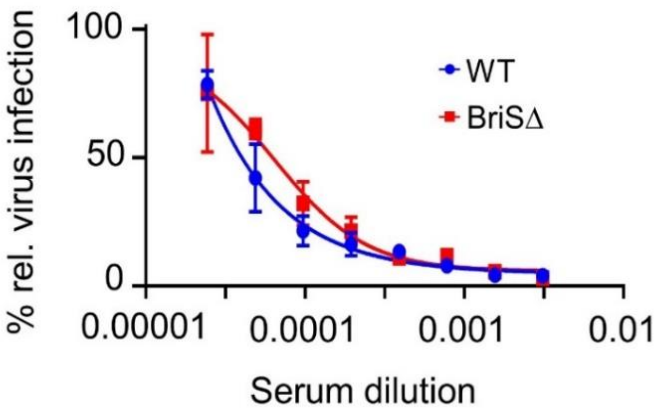

Supplementary Fig. 3: Human convalescent serum efficiently neutralizes WT and BriSΔ viruses. Serum dilutions as indicated were utilized for virus neutralization using Vero E6 cells. WT: SARS-CoV-2 wildtype; BriSΔ: SARS-CoV-2 S deletion variant. Data are presented as mean values with error bars showing +/- SD. N=3 biological replicates.

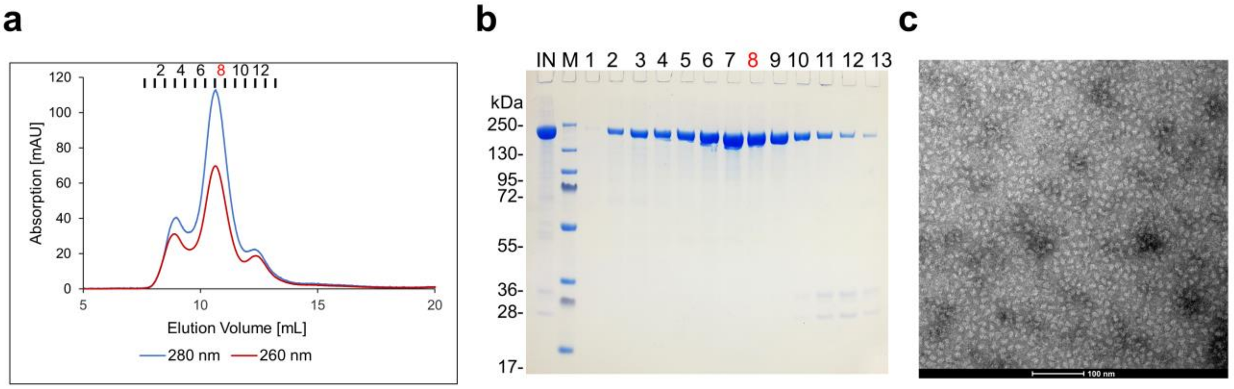

Supplementary Fig. 4: Purification and quality control of the BriSΔ glycoprotein. a Size-exclusion chromatography (SEC) of affinity-purified BriSΔ spike protein using a Superdex 200 column. Absorption was detected at 280 nm (blue line) and 260 nm (red line). Peak fractions are indicated. **b** SDS PAGE analysis of the SEC fractions from panel A. lane 1: input fraction, lane 2: molecular weight marker, lanes 3-15: fractions 1 to 13 from SEC. **c** Negative stain EM micrograph of SEC fraction 8 (scale bar: 100 nm). Preparations were performed 4 times (n=4).

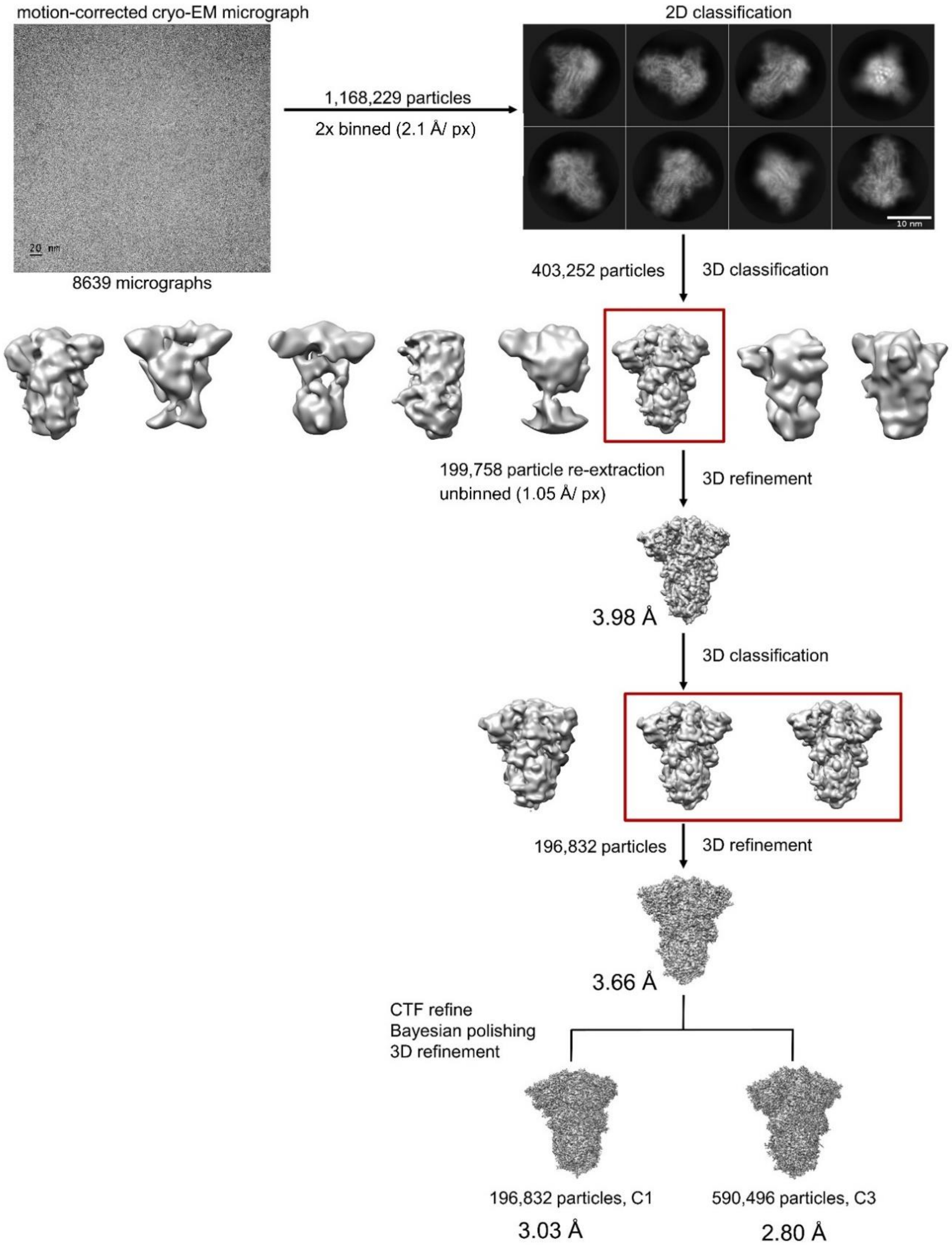

Supplementary Fig. 5: Cryo-EM image processing workflow. A motion-corrected cryo-EM micrograph is shown (scale bar 20 nm, particles circled in red), reference-free 2D class averages (scale bar 10 nm), 3D classifications and refinements resulting in a not symmetrized C1 and a C3-symmetrized cryo-EM map.

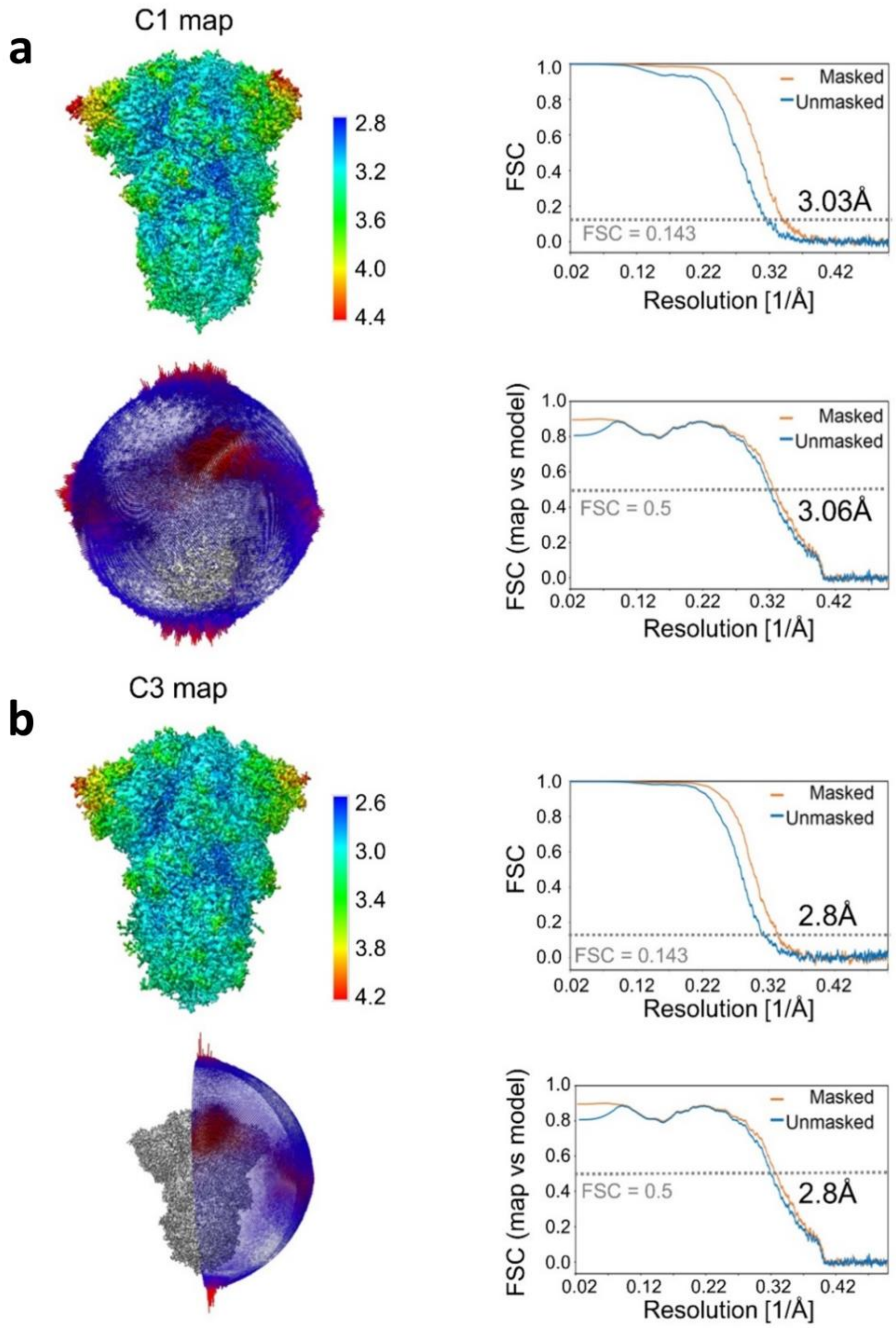

Supplementary Fig. 6: Cryo-EM structure validation. Top left: Cryo-EM reconstruction colored according to the local resolution from a side view. Top right: Fourier Shell Correlation (FSC) curve after gold standard refinement. Below left: Orientation distribution of views that contributed to this map. Longer red rods represent orientations that comprise more particles. Below right: Cross-validation FSC curves for the refined model versus the final masked and unmasked maps, shown for **a**. the unsymmetrized C1 map and **b**. the C3-symmetrized map.

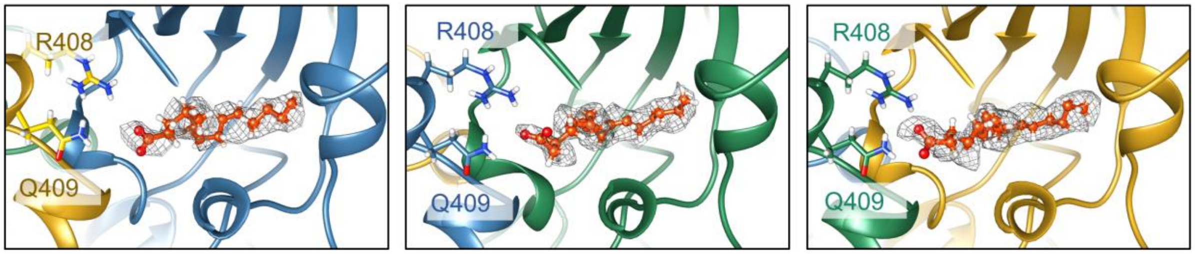

Supplementary Fig. 7: BriSΔ RBD structural organization. LA binding in each bipartite free fatty acid binding pocket in the unsymmetrized C1 structure is shown. EM density is shown as grey mesh.

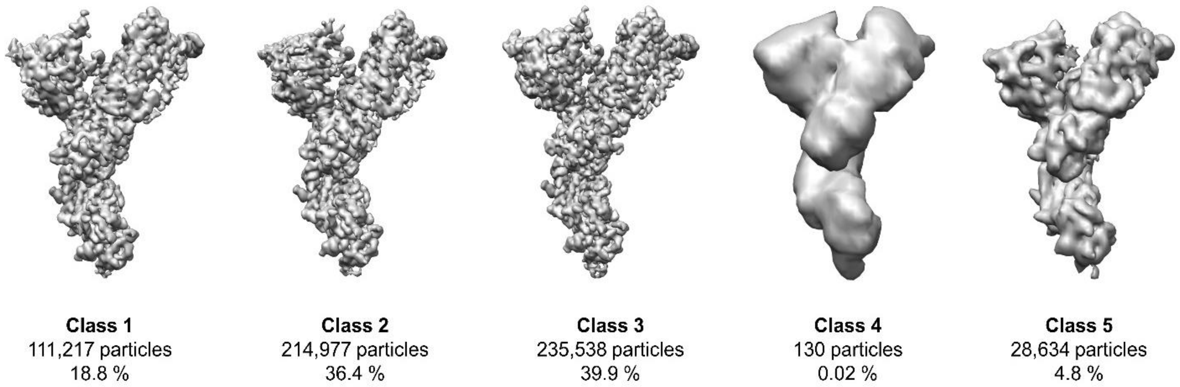

Supplementary Fig. 8: Masked 3D classification. Masked 3D classification focusing of individual chains within the S trimer into 5 classes. Class 1-3 comprised 95% of all particles. These classes all present LA-bound RBDs. The other 3D classes did not reach sufficient resolution to determine the presence or absence of LA in the RBD reconstructions.

a

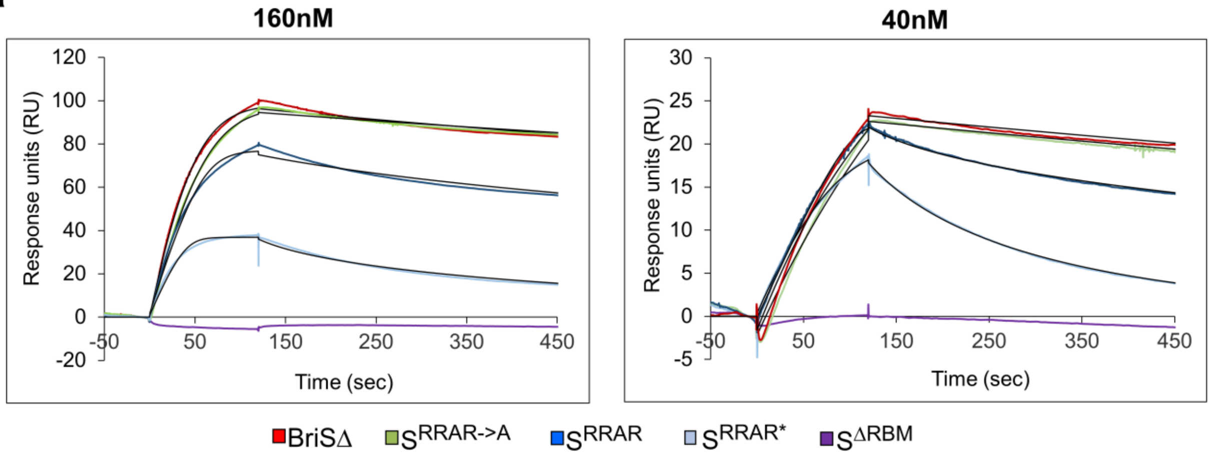

b

	K_D (nM)	k_{on} M^{-1} s^{-1}	k_{off} = s^{-1}
BriSΔ	1.8 ± 0.34	2.22 ± 0.49 x 10^5	3.9 ± 0.4 x 10^{-4}
$S^{RRAR\text{->}A}$	1.4 ± 0.3	2.5 ± 1.2 x 10^5	3.6 ± 2.2 x 10^{-4}
S^{RRAR}	2.51 ± 0.06	4.55 ± 0.04 x 10^5	11.4 ± 0.28 x 10^{-4}
S^{RRAR*}	12.2 ± 4.36	6.33 ± 2.18 x 10^5	70.69 ± 5.94 x 10^{-4}

Supplementary Fig. 9: Spike proteins binding to ACE2 by Surface Plasmon Resonance (SPR). a. Spike proteins BriSΔ, $S^{RRAR\text{->}A}$, S^{RRAR} and furin-cleaved S^{RRAR*} were analyzed for binding to ACE2 receptor immobilized on a streptavidin-coated sensor chip. Sensorgrams for representative concentrations (160 nM and 40 nM) are shown including $S^{\Delta RBM}$ lacking the receptor-binding motif as control. K_D values, k_{on} and k_{off} are listed in **b**. Values for $S^{RRAR\text{->}A}$ are from [1].

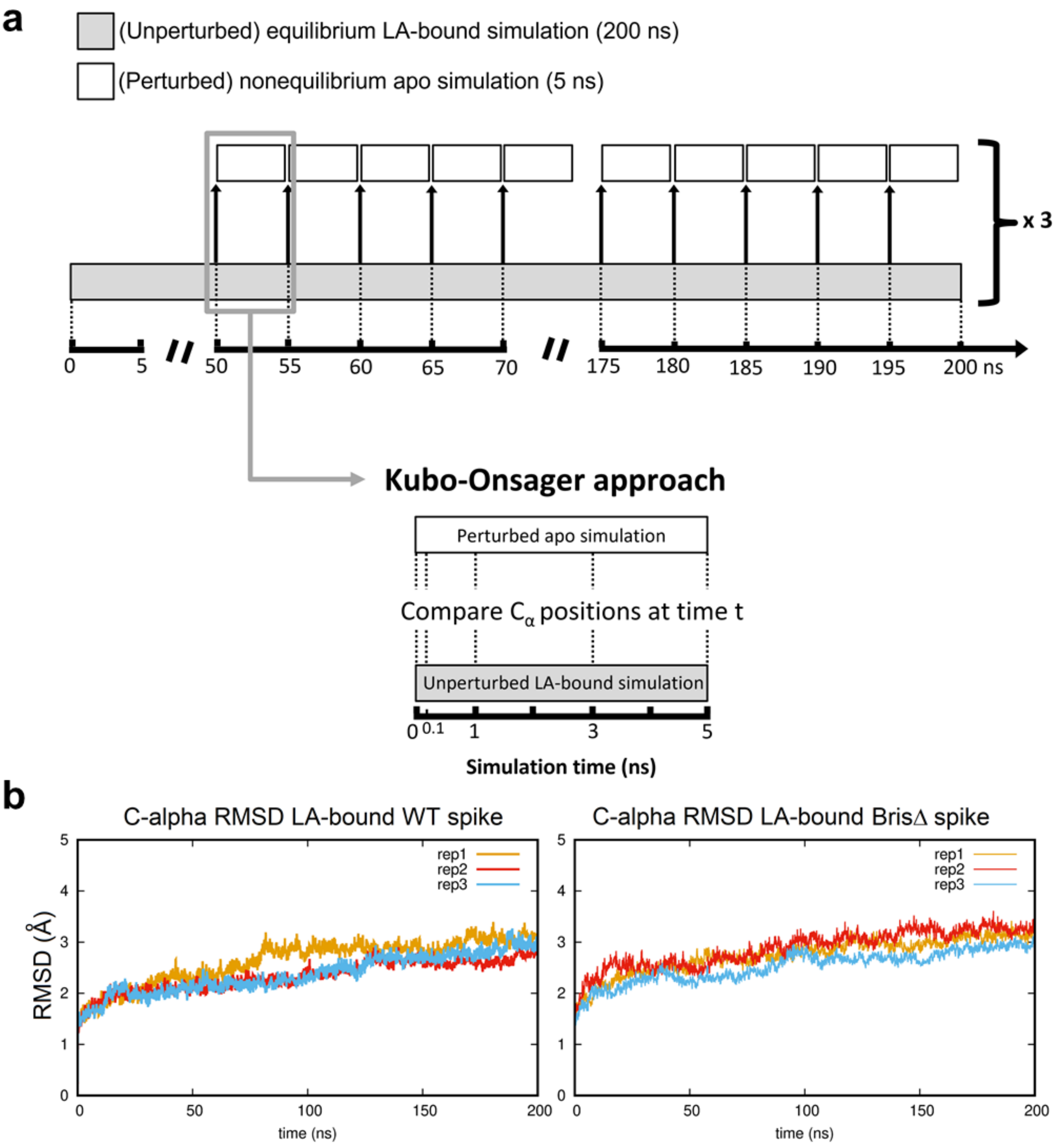

Supplementary Fig. 10: Nonequilibrium simulations of wildtype S and BriSΔ proteins. a. A schematic description of the procedure used to set up and analyze the nonequilibrium MD simulations. From the cryo-EM structure of the S proteins with LA bound, three equilibrium MD simulations, 200 ns each, were performed. These equilibrium simulations (indicated by rectangles filled in grey) were used to generate starting conformations for the nonequilibrium simulations (rectangles filled in white). **b.** shows the RMSDs

of the equilibration runs for WT and BriSΔ used as the start point for the nonequilibrium MD runs. The systems were considered sufficiently equilibrated after 50 ns and conformations were extracted every five nanoseconds beyond 50 ns from each LA-bound simulation (from 50-200 ns), and LA was removed. Each nonequilibrium simulation was run for five nanoseconds. The Kubo-Onsager approach [2-5] was used to extract the response of the system to LA removal (bottom panel). For each pair of unperturbed LA-bound and perturbed apo simulations, the positional deviations of each Cα at equivalent times (namely 0, 0.1, 1, 3 and 5 ns) were determined and averaged over all 90 simulations.

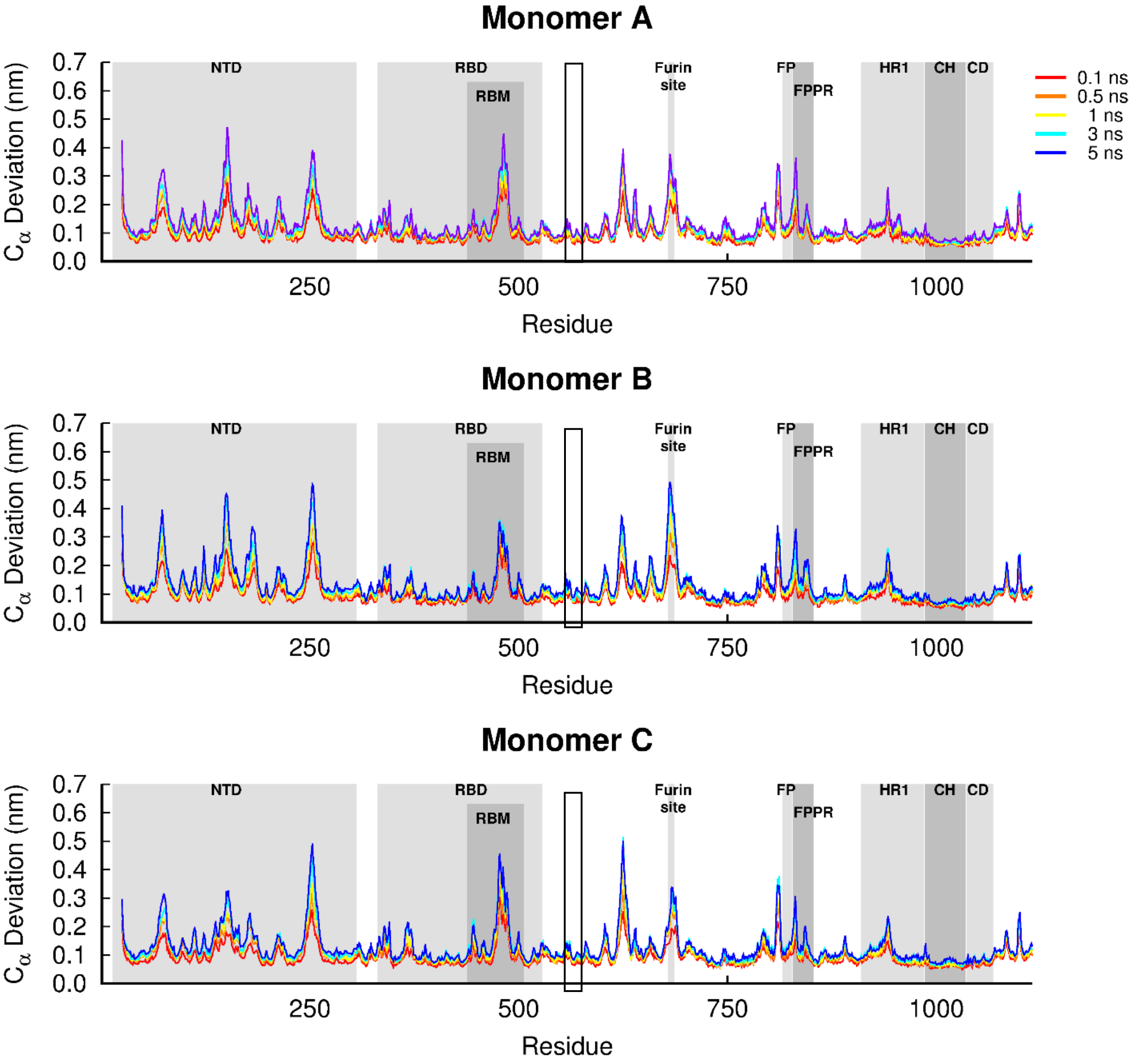

Supplementary Fig. 11: Average Cα-positional deviation in the five nanoseconds after removing LA from wildtype S protein. The average deviations were calculated using the Kubo-Onsager approach for the pairwise comparison between the nonequilibrium apo and equilibrium LA-bound simulations. The positions of important structural motifs are highlighted in grey, namely N-terminal domain (NTD), receptor-binding domain (RBD), receptor-binding motif (RBM), fusion peptide (FP), fusion peptide proximal region (FPPR), heptad repeat 1 (HR1), central helix (CH) and connector domain (CD). The V622-L629 region adjacent to R634 is boxed in black.

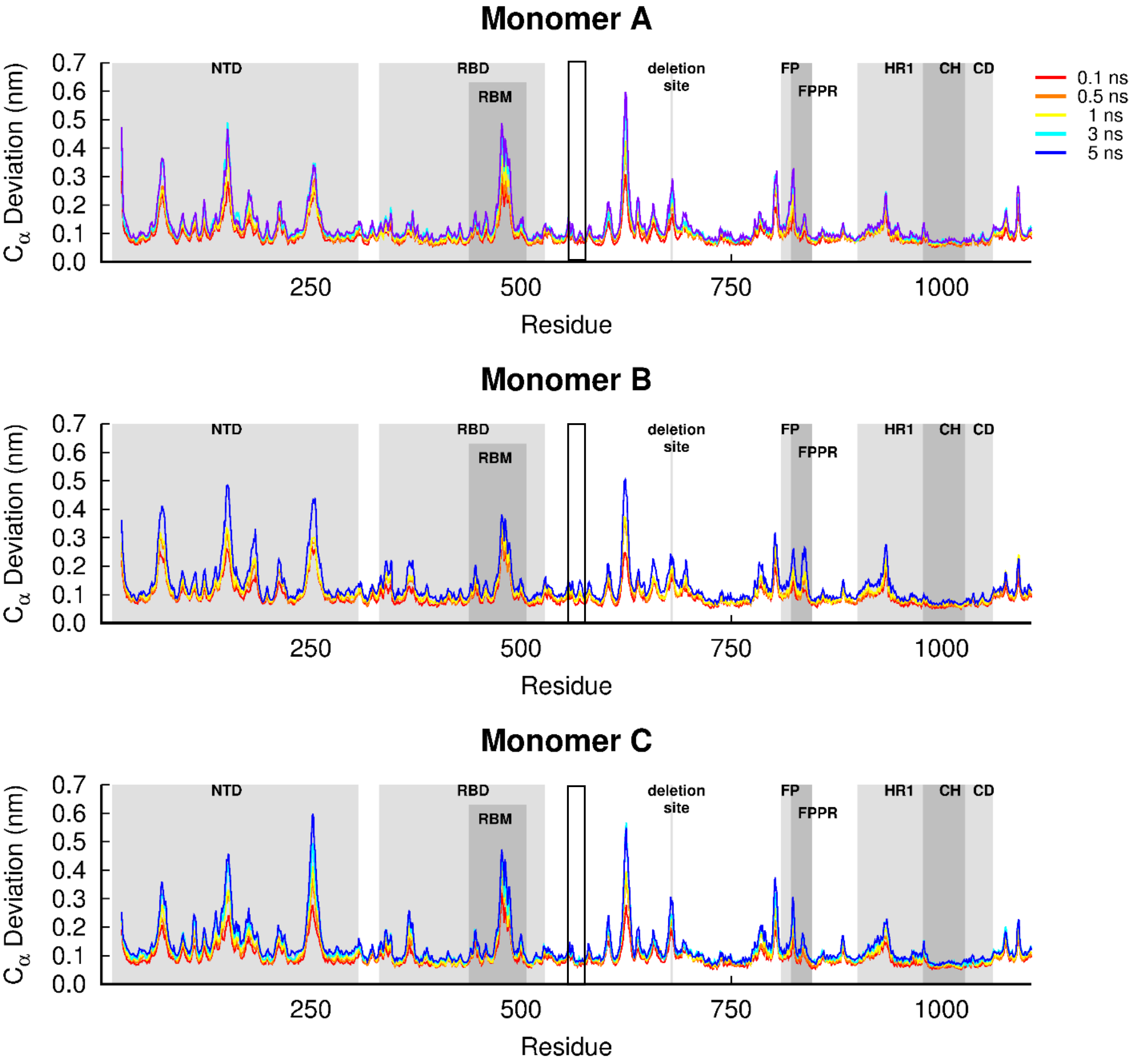

Supplementary Fig. 12: Average Cα-positional deviation in the five nanoseconds after removing LA from BriSΔ protein. The average deviations were calculated using the Kubo-Onsager approach for the pairwise comparison between the nonequilibrium apo and equilibrium LA-bound simulations. The positions of relevant structural motifs are highlighted in grey, namely the N-terminal domain (NTD), receptor-binding domain (RBD), receptor-binding motif (RBM), fusion peptide (FP), fusion peptide proximal region (FPPR), heptad repeat 1 (HR1), central helix (CH), connector domain (CD). The V622-L629 region adjacent to R634 is boxed in black.

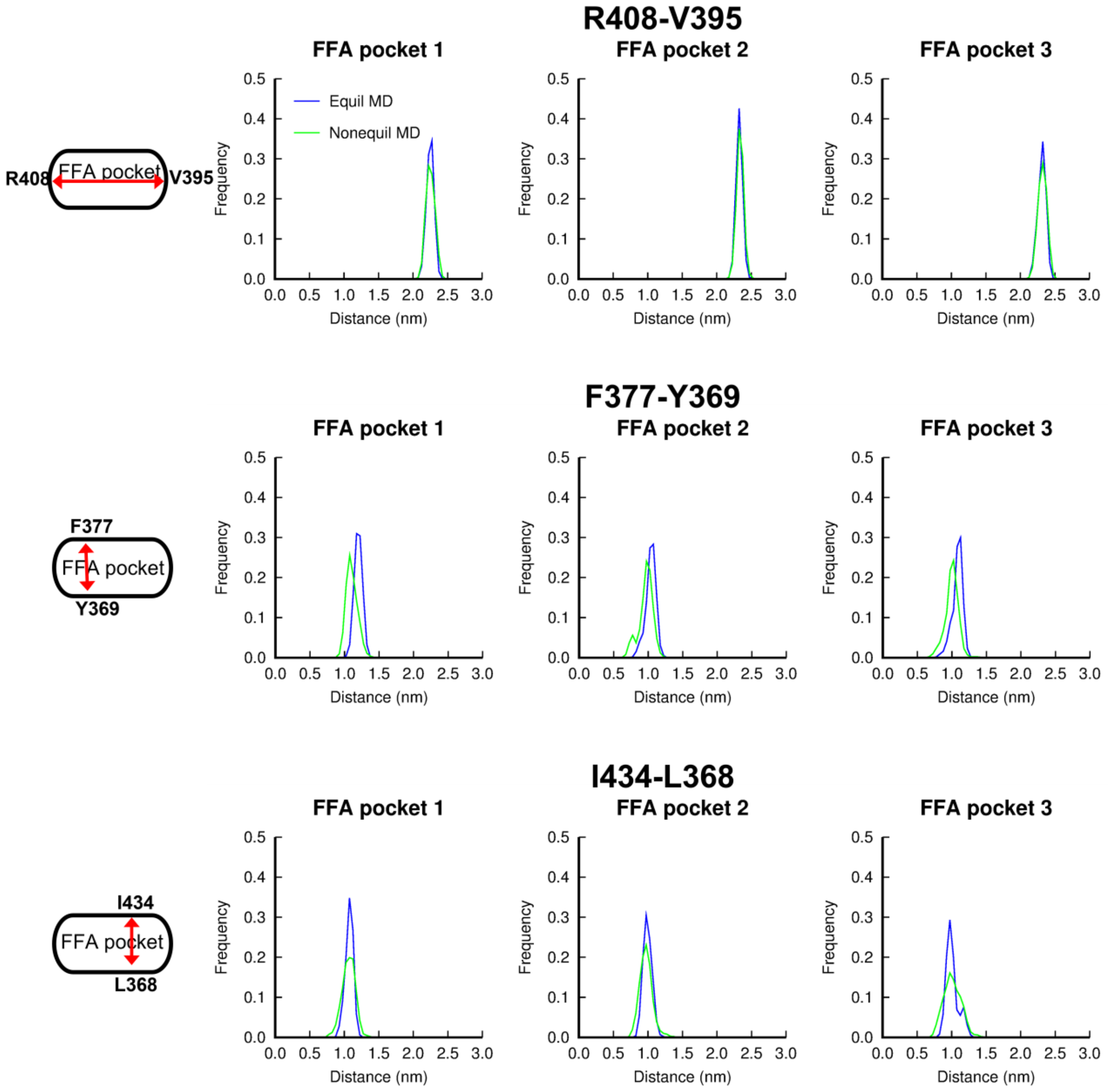

Supplementary Fig. 13: Distributions of the distances between R408-V395, F377-Y369 and I434-L368 in WT. Overall distribution of the distance between the center of mass of R408 and V395, F377 and Y369 and I434 and L368 in the equilibrium and nonequilibrium WT simulations. The histograms contain the distances for all three FFA pockets.

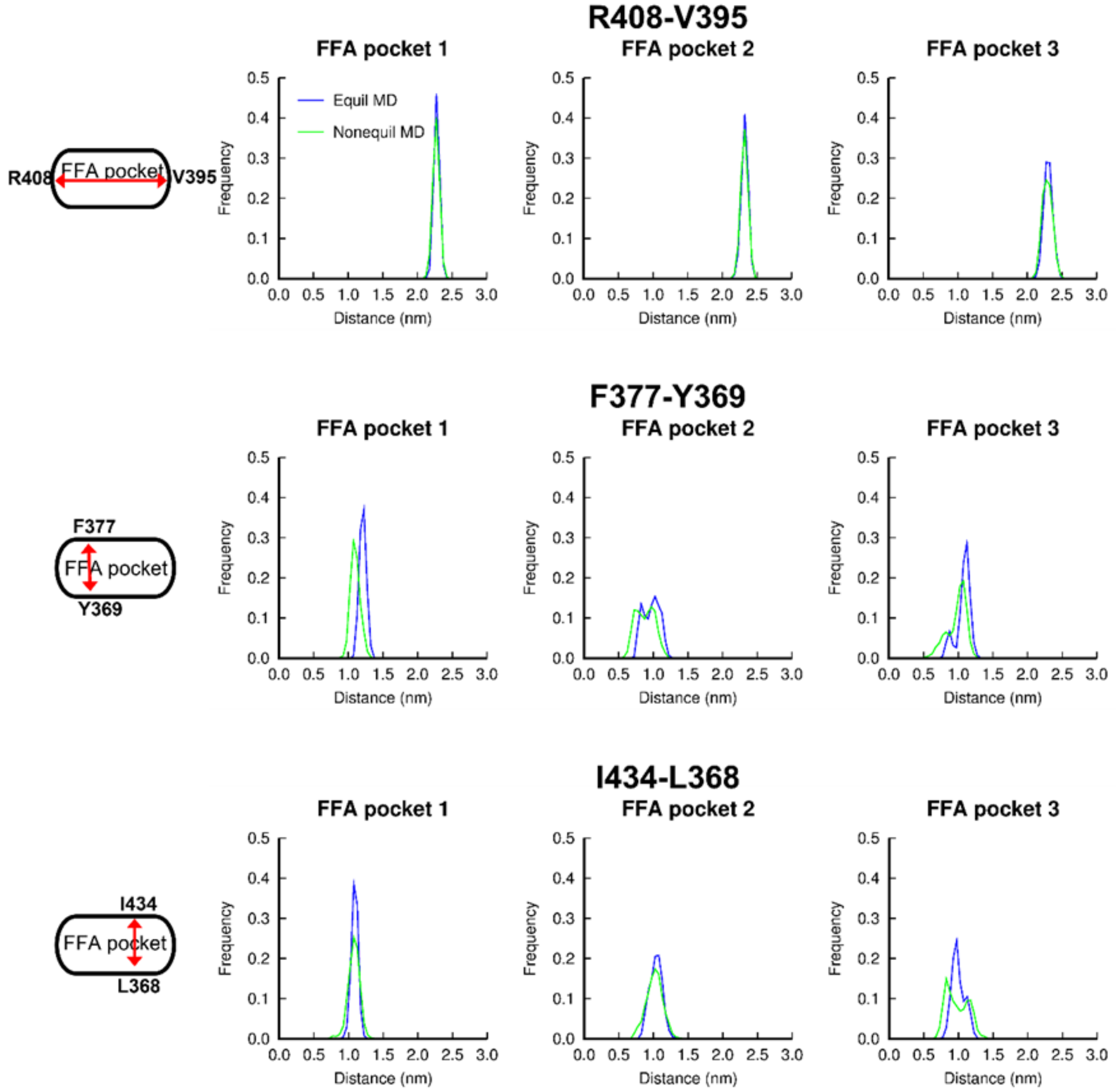

Supplementary Fig. 14: Distributions of the distances between R408-V395, F377-Y369 and I434-L368 in BriSΔ. Overall distribution of the distance between the center of mass of R408 and V395, F377 and Y369 and I434 and L368 in the equilibrium and nonequilibrium BriSΔ simulations. The histograms contain the distances for all three FFA pockets.

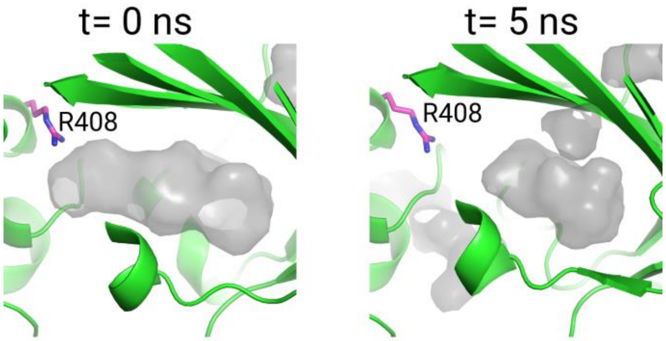

Supplementary Fig. 15: **Example of the FFA pocket in the beginning (t= 0 ns) and end (t= 5 ns) of a nonequilibrium simulation of BriSΔ**. Note the clear volume reduction of the pocket after 5 ns.

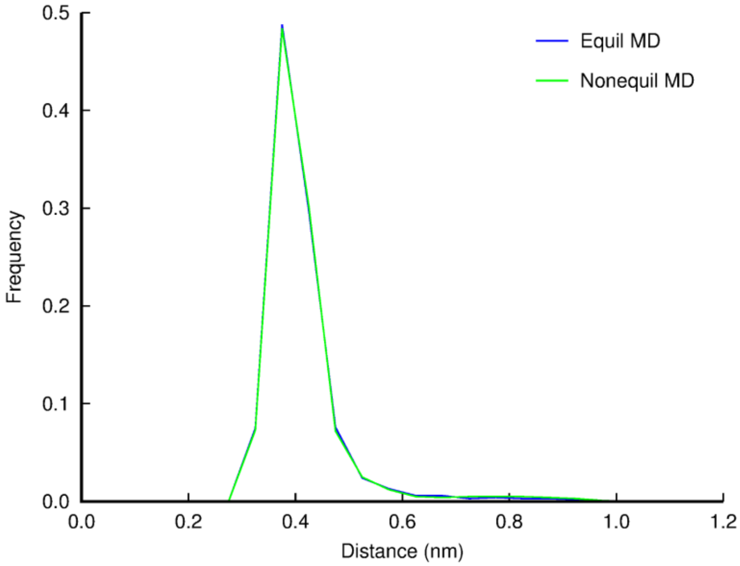

Supplementary Fig. 16: Distribution of the distance between R634 and Y837 in BriSΔ. Overall distribution of the distance between the positively charged sidechain of R634 and the aromatic sidechain of Y837 in the equilibrium and nonequilibrium MD simulations of BriSΔ. The histograms reflect the distances over the three chains.

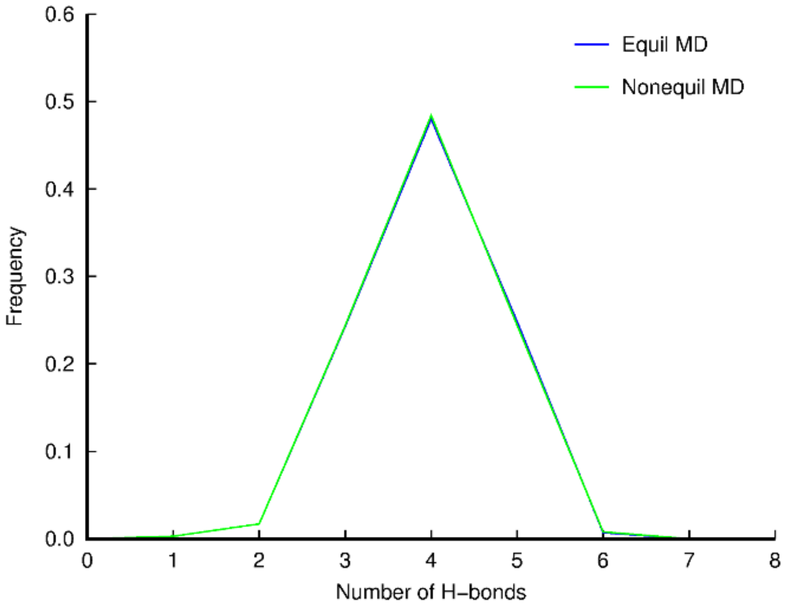

Supplementary Fig. 17: Number of hydrogen bonds between the R1039 and the protein in BriSΔ. Overall distribution of the number of hydrogen bonds formed by R1039 in the equilibrium and nonequilibrium MD simulations of BriSΔ. The histograms reflect the distances over the three chains.

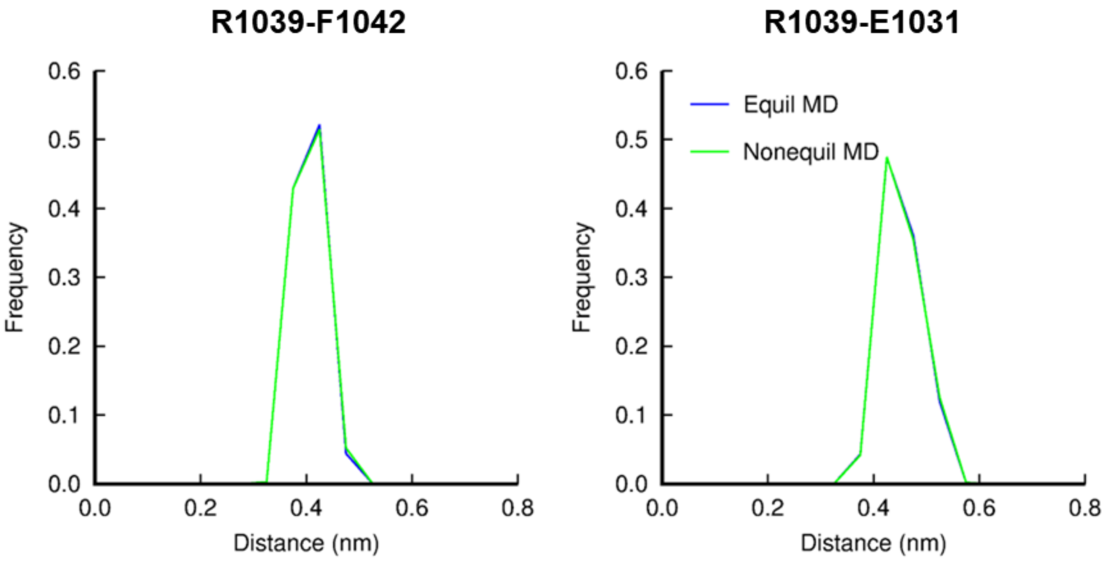

Supplementary Fig. 18: Distribution of the R1039-F1042 and R1039-E1031 distances in BriSΔ. Overall distribution of the distance between the sidechains of R1039, F1042 and E1031 in the equilibrium and nonequilibrium MD simulations of BriSΔ. The histograms reflect the distances over the three chains.

Supplementary Table 1: Cryo-EM data collection and refinement statistics.

	C1	C3
**Data collection and processing**		
Voltage (kV)	200	200
Magnification (nominal)	130,000	130,000
Pixel size (Å / px)	1.05 (0.525)	1.05 (0.525)
Flux (e^- /pix/sec)	6.4	6.4
Frames per exposure	55	55
Exposure (e^- / Å^2)	63.8	63.8
Defocus range (µm)	-0.8 to -2.0	-0.8 to -2.0
Micrographs collected	9,519	9,519
Particles final	196,832	590,496
Map sharpening B-factor (Å^2)	-97.6	-106.7
Masked resolution at 0.143 FSC (Å)	3.03	2.8
**Refinement**		
Composition		
Amino acids	3066	3036
Glycans	30	33
Ligands	3	3
RMSD bonds (Å)	0.003	0.004
RMSD angles (º)	0.564	0.555
Mean B-factors (Å^2)		
Amino acids	18.75	29.26
Ligands	33.61	41.97
Ramachandran		
Favored (%)	94.06	95.22
Allowed (%)	5.84	4.78
Outliers (%)	0.1	0.00
Rotamer outliers (%)	0.64	0.23
Clash score	2.77	2.49
C-beta outliers (%)	0.00	0.00
CaBLAM outliers (%)	3.14	2.51
CC (mask)	0.82	0.80
MolProbity score	1.46	1.36
EMRinger score	3.77	3.95
Model resolution (Å), 0.5 FSC threshold	3.0	2.8

Supplementary Table 2: N-linked glycosylation sites in recombinant SARS-CoV-2 S proteins.

WT SARS-CoV-2 S*	Recombinant SARS-CoV-2 S expressed in		
**Predicted (UNIPROT)**	**Freestyle 293F**** [6]	**Hi5**** [1]	**Hi5**** **(BriSΔ, this study)**
N_{17}LT		N_{17}LT	
N_{61}VT	N_{61}VT	N_{61}VT	N_{61}VT
N_{74}GT			
N_{121}NA[#]			
N_{122}AT	N_{122}AT	N_{122}AT	N_{122}AT
N_{149}KS			
N_{165}CT	N_{165}CT	N_{165}CT	N_{165}CT
N_{234}IT	N_{234}IT	N_{234}IT	N_{234}IT
N_{282}GT	N_{282}GT	N_{282}GT	N_{282}GT
N_{331}IT	N_{331}IT	N_{331}IT	N_{331}IT
N_{343}AT	N_{343}AT	N_{343}AT	N_{343}AT
N_{370}SA[#]			
N_{603}TS	N_{603}TS		
N_{616}CT	N_{616}CT	N_{616}CT	N_{616}CT
N_{657}NS	N_{657}NS		N_{657}NS
N_{709}NS	N_{709}NS	N_{706}NS	N_{709}NS
N_{717}FT	N_{717}FT	N_{714}FT	N_{717}FT
N_{801}FS	N_{801}FS	N_{798}FS	N_{801}FS
N_{1074}FT	N_{1074}FT	N_{1071}FT	N_{1074}FT
N_{1098}GT	N_{1098}GT	N_{1095}GT	N_{1098}GT
N_{1134}NT	N_{1134}NT	N_{1131}NT	N_{1134}NT
N_{1158}HT			
N_{1173}AS			
N_{1194}ES			

***** *YP_009724390.1*

***Sites lacking glycosylation in cryo-EM maps are omitted (boxes colored in grey).*

[#] *Predicted based on SARS-CoV-2 S protein* [6]

Supplementary Table 3: Alignment of SARS-CoV-2 S protein sequences used in this study

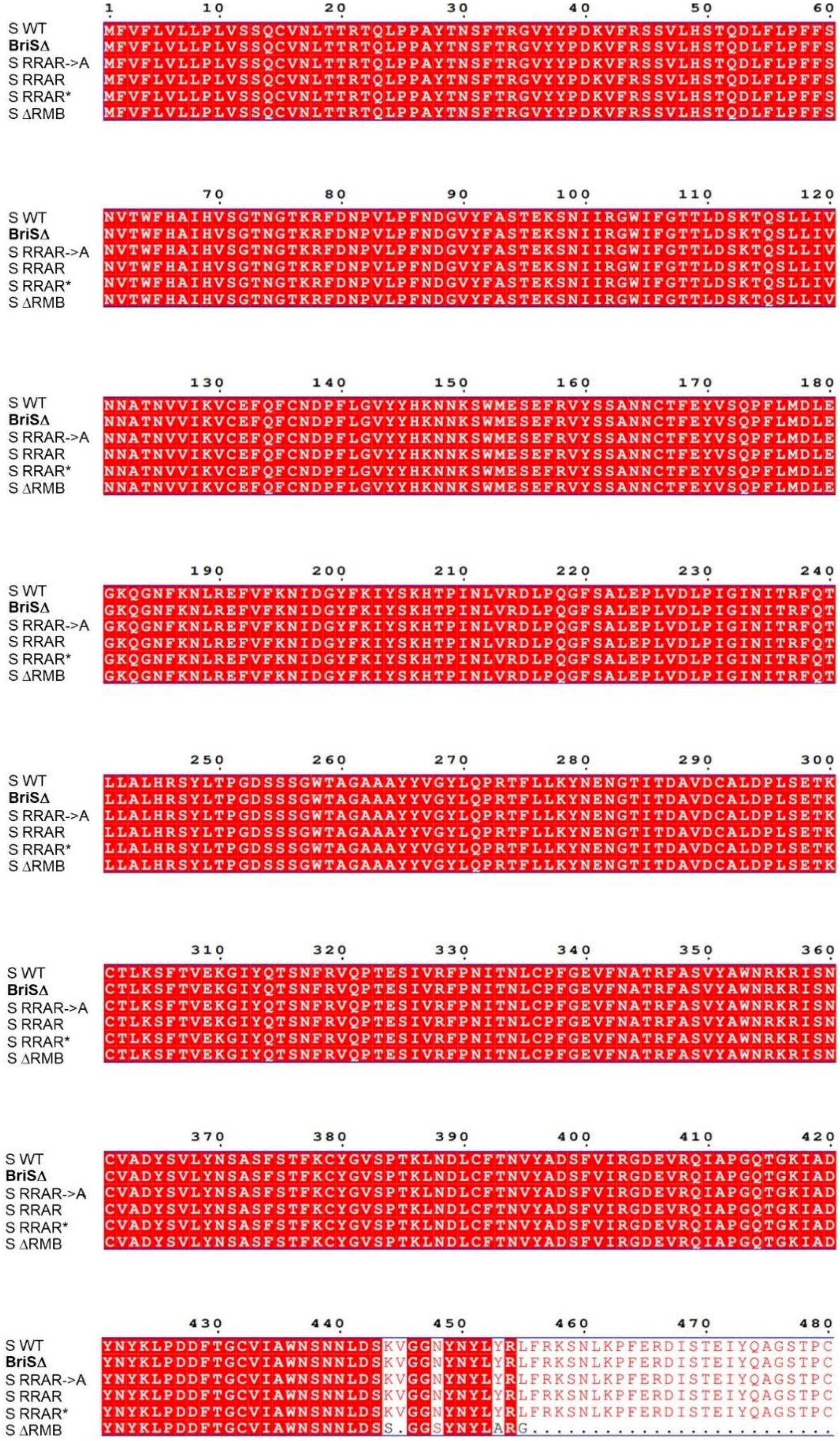

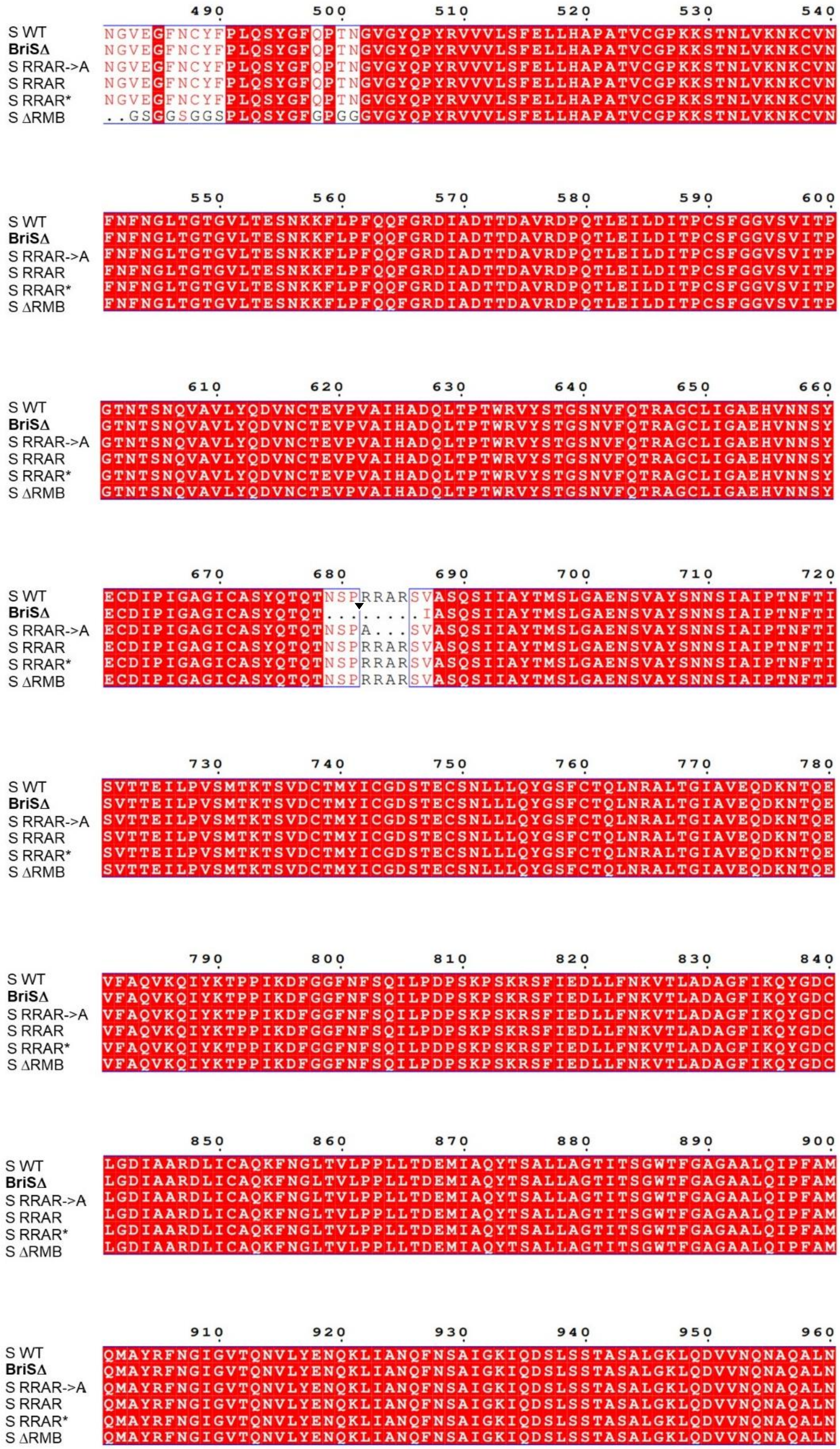

* *Furin cleavage in S^{RRAR*} is marked with a triangle filled in black*

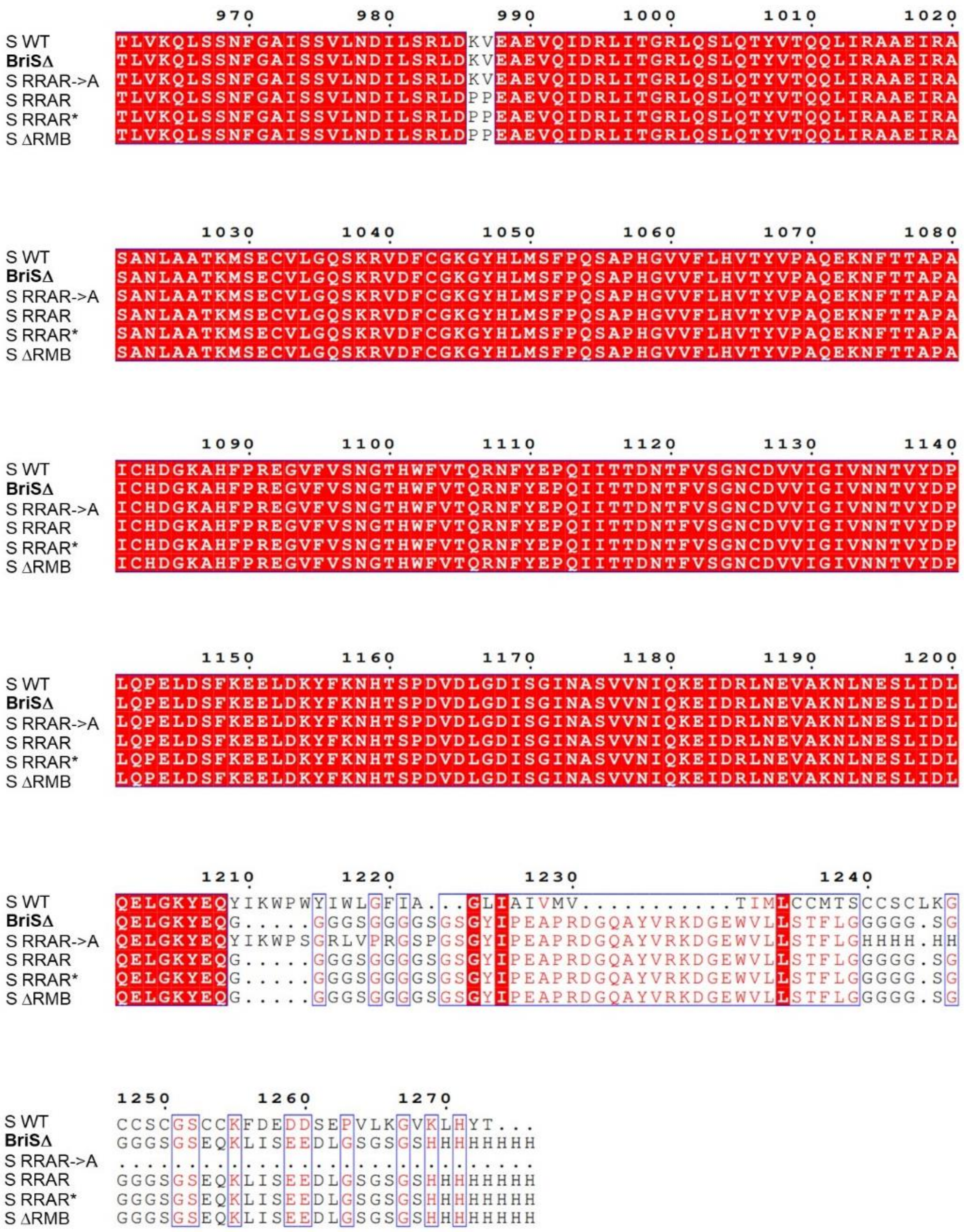

Supplementary Movie 1: An example of a targeted dynamics simulation. In this movie one of the 50 (10 ns) replicates shows the opening of the RBD of the WT apo SARS-CoV-2 S protein trimer when 0.2 kJ/mol/nm force constant was applied to atoms of the RBD to pull it from a starting, closed, equilibrated (following 200 ns simulation) conformation to the raised RBD position in EMD-1114.

References

1. Toelzer, C. et al. Free fatty acid binding pocket in the locked structure of SARS-CoV-2 spike protein. *Science* **370**, 725-730 (2020).

2. Ciccotti, G., Jacucci, G. & McDonald, I.R. Thought-experiments by molecular dynamics. *J Stat Phys* **21**, 1-12 (1979).

3. Ciccotti, G. Computer simulation in material science. *P. V. Meyer M, Ed. (Kluwer Academic Publishers)*, 119–137 (1991).

4. Ciccotti, G. & Ferrario, M. Non-equilibrium by molecular dynamics: a dynamical approach. *Mol Simul* **42**, 1385-1400 (2016).

5. Oliveira, A.S.F., Ciccotti, G., Haider, S. & Mulholland, A.J. Dynamical nonequilibrium molecular dynamics reveals the structural basis for allostery and signal propagation in biomolecular systems. *Eur. Phys. J. B* **94**, 144 (2021).

6. Walls, A.C. et al. Structure, Function, and Antigenicity of the SARS-CoV-2 Spike Glycoprotein. *Cell* **181**, 281-292 e6 (2020).
