# Supplementary material for: Structural insights in cell-type specific evolution of intra-host diversity by SARS-CoV-2
Source: Nat Commun. 2022 Jan 11;13:222. doi: 10.1038/s41467-021-27881-6 (PMC8752678; doi:10.1038/s41467-021-27881-6)
Supplement: Supplementary file 3 — Description of Additional Supplementary Files [file 41467_2021_27881_MOESM3_ESM.pdf]

Description of Additional Supplementary Files

File Name: Supplementary Movie 1
Description: An example of a targeted dynamics simulation.
