# Supplementary material for: Structural insights in cell-type specific evolution of intra-host diversity by SARS-CoV-2
Source: Nat Commun. 2022 Jan 11;13:222. doi: 10.1038/s41467-021-27881-6 (PMC8752678; doi:10.1038/s41467-021-27881-6)
Supplement: Supplementary file 5 — Reporting Summary [file 41467_2021_27881_MOESM5_ESM.pdf]

nature portfolio

Corresponding author(s): Imre Berger, Christiane Schaffitzel, Kapil Gupta

Last updated by author(s): Dec 4, 2021

Reporting Summary

Nature Portfolio wishes to improve the reproducibility of the work that we publish. This form provides structure for consistency and transparency in reporting. For further information on Nature Portfolio policies, see our Editorial Policies and the Editorial Policy Checklist.

Statistics

For all statistical analyses, confirm that the following items are present in the figure legend, table legend, main text, or Methods section.

n/a	Confirmed	
☐	☒	The exact sample size (*n*) for each experimental group/condition, given as a discrete number and unit of measurement
☐	☒	A statement on whether measurements were taken from distinct samples or whether the same sample was measured repeatedly
☐	☒	The statistical test(s) used AND whether they are one- or two-sided<br>*Only common tests should be described solely by name; describe more complex techniques in the Methods section.*
☒	☐	A description of all covariates tested
☒	☐	A description of any assumptions or corrections, such as tests of normality and adjustment for multiple comparisons
☐	☒	A full description of the statistical parameters including central tendency (e.g. means) or other basic estimates (e.g. regression coefficient) AND variation (e.g. standard deviation) or associated estimates of uncertainty (e.g. confidence intervals)
☐	☒	For null hypothesis testing, the test statistic (e.g. *F*, *t*, *r*) with confidence intervals, effect sizes, degrees of freedom and *P* value noted<br>*Give P values as exact values whenever suitable.*
☒	☐	For Bayesian analysis, information on the choice of priors and Markov chain Monte Carlo settings
☒	☐	For hierarchical and complex designs, identification of the appropriate level for tests and full reporting of outcomes
☒	☐	Estimates of effect sizes (e.g. Cohen's *d*, Pearson's *r*), indicating how they were calculated

Our web collection on statistics for biologists contains articles on many of the points above.

Software and code

Policy information about availability of computer code

Data collection	We used EPU 3.10 for CryoEM data collection.<br>We used Analyst 1.7.0 for MS data collection.<br>We used Biacore T200 control software v1.0 for SPR data collection.
Data analysis	We used Cell Reporter Xpress for analysis of virus infected cells.<br>We used MotionCor2-1.1, Gctf1.18, Relion 3.0 and Relion 3.1 for CryoEM data processing.<br>We used MultiQuant 3.0.2 for MS data analysis.<br>We used Biacore Evaluation software v1.0 for SPR data analysis.<br>We used GROMACS-2019.2, VMD-1.9.1, Chimera-1.10.2 for Molecular dynamics simulation analysis.<br>We used Phenix 1.19.2-4158-000 suite and WinCoot 0.8.9.2 Structure model building and refinement.<br>Graphpad prism v8.4.3 for preparing graphs.

For manuscripts utilizing custom algorithms or software that are central to the research but not yet described in published literature, software must be made available to editors and reviewers. We strongly encourage code deposition in a community repository (e.g. GitHub). See the Nature Portfolio guidelines for submitting code & software for further information.

Data

Policy information about availability of data

All manuscripts must include a data availability statement. This statement should provide the following information, where applicable:

- Accession codes, unique identifiers, or web links for publicly available datasets
- A description of any restrictions on data availability
- For clinical datasets or third party data, please ensure that the statement adheres to our policy

Datasets and coordinates generated during the current study have been deposited in the Electron Microscopy Data Bank (EMDB) under accession numbers EMD-12818 (C3 structure) and EMD-12842 (C1 structure) and in the Protein Data Bank (PDB) under accession numbers: PDBID-7OD3 (C3 structure) and PDBID-7ODL (C1 structure).

Field-specific reporting

Please select the one below that is the best fit for your research. If you are not sure, read the appropriate sections before making your selection.

☒ Life sciences ☐ Behavioural & social sciences ☐ Ecological, evolutionary & environmental sciences

For a reference copy of the document with all sections, see nature.com/documents/nr-reporting-summary-flat.pdf

Life sciences study design

All studies must disclose on these points even when the disclosure is negative.

Sample size	Sample sizes were chosen based on what is currently done in the field.<br>No sample size pre-calculation was performed as it is not currently done in the field.<br>Experimental replicates (biological or technical) were performed as indicated in the figure legends and/or methods, which correspond to what is currently done in the field.
Data exclusions	No data was excluded.
Replication	Reproducibility was checked by performing experiments in replicates (either duplicates or triplicates unless mentioned in the figure legend specifically) and some of these are indicated in corresponding figure legends and/or methods. All the replications gave similar results.
Randomization	Randomization is not applicable because for each experiment all the conditions/samples were used at the same time (conditions/samples were always in same group).
Blinding	Investigators were not blinded because the type of data in this manuscript doesn't require blinding. Part of our data was generated through computer based analyses and other experimental data was either only qualitative (CryoEM structure, protein purification, MS) or was already quantitative (RT-PCR, SPR, cPASS).

Reporting for specific materials, systems and methods

We require information from authors about some types of materials, experimental systems and methods used in many studies. Here, indicate whether each material, system or method listed is relevant to your study. If you are not sure if a list item applies to your research, read the appropriate section before selecting a response.

Materials & experimental systems

n/a	Involved in the study	
☐	☒	Antibodies
☐	☒	Eukaryotic cell lines
☒	☐	Palaeontology and archaeology
☒	☐	Animals and other organisms
☒	☐	Human research participants
☒	☐	Clinical data
☒	☐	Dual use research of concern

Methods

n/a	Involved in the study	
☒	☐	ChIP-seq
☒	☐	Flow cytometry
☒	☐	MRI-based neuroimaging

Antibodies

Antibodies used	Primary Antibody:<br>Antibody against the SARS-CoV-2 nucleocapsid protein (N] (200-401-A50, Rockland); 1:2000 dilution; polyclonal.<br>Antibody recognizing the S protein receptor-binding domain (RBD) (Ab02013-10.159, Absolute Antibody); Clone Name: Sb#15.

	Secondary Antibody<br>Alexa Fluor 568: Goat anti-Rabbit IgG (H+L) Highly Cross-Adsorbed Secondary Antibody (A-11036, Life Technologies); 1:2000 dilution; polyclonal.<br>Alexa Fluor 488: Goat anti-Rabbit IgG (H+L) Cross-Adsorbed Secondary Antibody (A-11008, Thermofisher); 1:2000 dilution; polyclonal.
Validation	Antibody against the SARS-CoV-2 nucleocapsid protein (N) (200-401-A50, Rockland): Antibody generated in Rabbit against SARS-CoV Nucleocapsid (N) Protein; Application: ELISA, IF Microscopy, Flow Cytometry, Western Blot, Immunohistochemistry, Can bind to SARS-CoV-2 N protein (de Vries M et al., J Virol., 2021)<br><br>Antibody recognizing the S protein receptor-binding domain (RBD) (Absolute Antibody; Sb#15): Recombinant monoclonal antibody recognizing SARS-CoV-2 Spike RBD, manufactured with variable regions (i.e. specificity) from the synthetic nanobody Sb#15. The clone for Sb #15 was originally isolated in a form of a synthetic nanobody (sybody] via a 'target swap' selection procedure against RBD-vYFP using ribosomal display and against RBD-Fc fusion during phage display rounds (Walter et al, bioRxiv, 2020); Application: LISA, Binding to RBD of SARS-CoV-2, Inhibition of ACE2 binding of SARS-CoV-2.

Eukaryotic cell lines

Policy information about cell lines

Cell line source(s)	Human Calu-3 (ATCC ® HTB-55™).<br>Human Caco-2 (ATCC® HTB-37™).<br>African green monkey Vero E6 (ATCC® CRL 1586™).<br>Caco-2 cell line expressing ACE2 (Caco-2-ACE2): produced from the Caco-2 cells (ATCC® HTB-37™) by lentivirus transduction by Dr Yohei Yamauchi, University of Bristol a nd was a kind gift; ref: Daly, J.L. et al., Science 370, 861-865 (2020).<br>Vero E6 cells modified to constitutively express TMPRSS2 (Vero E6/TMPRSS2 cells5; obtained from NIBSC, UK).<br>Trichoplusia Ni (Hi5) cells (Cellosaurus Tni-FNL; CVCL_RY32)
Authentication	Already authenticated cell lines were purchased from ATCC/NIBSC as described. Cell lines were not authenticated further in our labs.
Mycoplasma contamination	Cell lines were not tested for Mycoplasma contamination.
Commonly misidentified lines<br>(See ICLAC register)	No commonly misidentified cell lines were used in this study.
